# Ethyl 2-(4-benzoyl-2,5-dimethyl­phen­oxy)acetate

**DOI:** 10.1107/S1600536809043190

**Published:** 2009-10-28

**Authors:** H. C. Devarajegowda, Shaukath Ara Khanum, S. Jeyaseelan, Waleed Al Eryani, J. Shylajakumari

**Affiliations:** a Department of Physics, Yuvaraja’s College (Constituent College), University of Mysore, Mysore 570 005, Karnataka, India; b Department of Chemistry, Yuvaraja’s College (Constituent College), University of Mysore, Mysore 570 005, Karnataka, India; c Department of Physics, AVK College for Women, Hassan 573 201, Karnataka, India

## Abstract

The title compound, C_19_H_20_O_4_, was synthesized *via* a Fries rearrangement of hydr­oxy benzophenone. The dihedral angle between the least-squares planes of the two benzene rings is 69.04 (11)°. The mol­ecular structure displays an intra­molecular non-classical C—H⋯O hydrogen bond.

## Related literature

hydr­oxy benzophenones may obtained from natural products, see: Henry *et al.* (1999 ▶); Vidya *et al.* (2003 ▶); Cuesta-Rubio *et al.* (2002 ▶) and by synthetic methods, see: Hsieh *et al.* (2003 ▶); Revesz *et al.* (2004 ▶); Schlitzer *et al.* (2002 ▶). For their biological activity, see: Jiri *et al.* (1991 ▶); Palomer *et al.* (2000 ▶, 2002 ▶); Palaska *et al.* (2002 ▶); Khanum *et al.* (2004*a* ▶,*b* ▶). Benzophenone analogues with nitro substituents exhibit significant *in vivo* antitumor activity and they have been reported to show activity as immunomodulators, see: Leonard (1997 ▶). Nitro benzophenone derivatives show strong cytotoxic activity while the corresponding aminobenzophenone derivatives show weak activity, see: Kumazawa *et al.* (1997 ▶). For the antimicobial activity of benzophenone derivatives, see: Selvi *et al.* (2003 ▶).
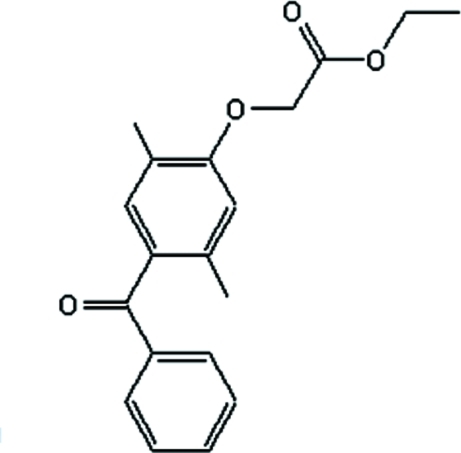


## Experimental

### 

#### Crystal data

C_19_H_20_O_4_
*M* *_r_* = 312.35
Triclinic, 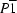

*a* = 8.148 (4) Å
*b* = 8.635 (4) Å
*c* = 13.029 (7) Å
α = 84.054 (8)°
β = 81.176 (8)°
γ = 66.559 (7)°
*V* = 830.2 (7) Å^3^
*Z* = 2
Mo *K*α radiation
μ = 0.09 mm^−1^
*T* = 295 K
0.21 × 0.20 × 0.10 mm

#### Data collection

Bruker SMART CCD diffractometer
Absorption correction: multi-scan (*SADABS*; Sheldrick, 2004 ▶) *T*_min_ = 0.982, *T*_max_ = 0.991
8847 measured reflections
3391 independent reflections
2630 reflections with *I* > 2σ(*I*)
*R*_int_ = 0.025

#### Refinement

*R*[*F* ^2^ > 2σ(*F* ^2^)] = 0.057
*wR*(*F* ^2^) = 0.173
*S* = 1.05
3391 reflections
209 parameters
H-atom parameters constrained
Δρ_max_ = 0.36 e Å^−3^
Δρ_min_ = −0.25 e Å^−3^

### 

Data collection: *SMART* (Bruker, 2001 ▶); cell refinement: *SAINT* (Bruker, 2001 ▶); data reduction: *SAINT*; program(s) used to solve structure: *SHELXS97* (Sheldrick, 2008 ▶); program(s) used to refine structure: *SHELXL97* (Sheldrick, 2008 ▶); molecular graphics: *ORTEP-3* (Farrugia, 1999 ▶); software used to prepare material for publication: *SHELXL97*.

## Supplementary Material

Crystal structure: contains datablocks global, I. DOI: 10.1107/S1600536809043190/rk2174sup1.cif

Structure factors: contains datablocks I. DOI: 10.1107/S1600536809043190/rk2174Isup2.hkl

Additional supplementary materials:  crystallographic information; 3D view; checkCIF report

## Figures and Tables

**Table 1:** Hydrogen-bond geometry (Å, °)

*D*—H⋯*A*	*D*—H	H⋯*A*	*D*⋯*A*	*D*—H⋯*A*
C15—H15*A*⋯O2	0.96	2.28	2.751 (3)	110

**Fig. 1.**
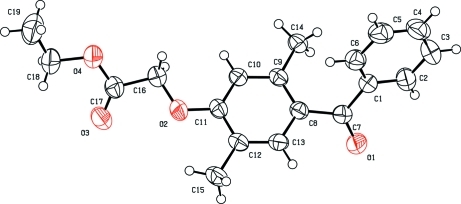
The asymmetric unit of the title compound with the atom-numbering scheme. Displacement ellipsoids are drawn at the 50% probability level. H atoms are presented as a small spheres of arbitrary radius.

## supplementary crystallographic information

### Comment

Hydroxy benzophenones are achieved from natural products (Henry *et al.*, 1999; Vidya *et al.*, 2003; Cuesta-Rubio *et al.*, 2002) as well as by synthetic methods (Hsieh *et al.*, 2003; Schlitzer *et al.*, 2002; Revesz *et al.*, 2004). The great importance of these substances is essentially due to the diverse biological and chemical properties they acquire. Benzophenone analogues possess a high analgesic (Jiri *et al.*, 1991) efficacy and also endowed with anti-inflammatory property (Palomer *et al.*, 2000; Palomer *et al.*, 2002; Palaska *et al.*, 2002; Khanum *et al.*, 2004a,b).

Benzophenone analogues with nitro substituent exhibit significant *in vivo* antitumor activity and they have been reported to show activity as immunomodulators (Leonard, 1997). Based on these report, *in vitro* and *in vivo* studies of a series of novel nitro- and amino-substituted benzophenones have been investigated as potential anticancer agents. Nitro benzophenone derivative showed strong cytotoxic activity while the corresponding aminobenzophenone derivatives showed weak activity (Kumazawa *et al.*, 1997). Besides benzophenone derivatives endowed with anti-microbial activity, for instance isoprenylated benzophenone, at its lower concentration of 500 to 1000 p.p.m. inhibits aflatoxin production in *Aspergillus flavus*, relatively greater than inhibition growth of the fungus  - Selvi *et al.*, (2003).

The molecular structure of title compound is shown on Fig.1. The dihedral angle between least-squares planes (two phenyl rings) is 69.04 (11)°. The intramolecular non-classical C–H···O hydrogen bond (Table 1) is observed.

### Experimental

2,5-Dimethyl phenyl benzoate was synthesized from 2,5-dimethyl phenol and benzoyl chloride in presence of 10% sodium hydroxide. 4-Hydroxy-2,5-dimethyl benzophenone was achieved from 2,5-dimethyl phenyl benzoate by Fries rearrangement.

In a typical procedure, a mixture of 4-hydroxy-2,5-dimethylbenzophenone (4.5 g, 0.02 mol) and ethylchloroacetate (2.4 g, 0.02 mol) in dry acetone (60 ml) and anhydrous potassium carbonate (2.8 g, 0.02 mol) was refluxed for 6 h then cooled and the solvent removed under reduced pressure. The residual mass was triturated with ice water to remove potassium carbonate and extracted with ether (3 × 50ml) and the ether layer was washed with 10% sodium hydroxide solution (3 × 30ml) followed by water (3 × 30ml) and then dried over anhydrous sodium sulfate and evaporated to dryness to get crude solid, which on recrystallization with ethanol gave 4-benzoyl-2,5-dimethyl phenoxy ethyl acetate.

M.p. 329 K; IR (Nujol): 1740 (ester, C═O), 1665 cm^-1^ (C═O). ^1^H NMR (CDCl_3_): δ 1.2 (t, J=7 Hz, 3H, CH_3_ of ester), 2.2-2.3 (d, 6H, 2*Ar*-CH_3_), 4.25 (q, J=6 Hz, 2H, CH_2_ of ester), 4.45 (s, 2H, OCH_2_), 7.2-7.8 (bm, 7H, *Ar*–H); Anal. Cal. for C_19_H_20_O_4_: C, 72.61%; H, 6.36%. Found: C, 72.29%; H, 6.15%.

### Refinement

All H atoms were positioned at calculated positions with C–H = 0.93Å for aromatic H, 0.97Å for methylene H and 0.96Å for methyl H, and refined using a riding model with *U*_iso_(H) = 1.5*U*_eq_(C) for methyl H and *U*_iso_(H) = 1.2*U*_eq_(C) for other.

### Crystal data

**Table:**

C_19_H_20_O_4_	*Z* = 2
*M**_r_* = 312.35	*F*(000) = 332
Triclinic, *P*1	*D*_x_ = 1.250 Mg m^−^^3^
Hall symbol: -P 1	Melting point: 329 K
*a* = 8.148 (4) Å	Mo *K*α radiation, λ = 0.71073 Å
*b* = 8.635 (4) Å	Cell parameters from 3391 reflections
*c* = 13.029 (7) Å	θ = 1.6–26.4°
α = 84.054 (8)°	µ = 0.09 mm^−^^1^
β = 81.176 (8)°	*T* = 295 K
γ = 66.559 (7)°	Plate, colourless
*V* = 830.2 (7) Å^3^	0.21 × 0.20 × 0.10 mm

### Data collection

**Table:**

Bruker SMART CCD diffractometer	3391 independent reflections
Radiation source: fine-focus sealed tube	2630 reflections with *I* > 2σ(*I*)
graphite	*R*_int_ = 0.025
ω and φ scans	θ_max_ = 26.4°, θ_min_ = 1.6°
Absorption correction: multi-scan (*SADABS*; Sheldrick, 2004)	*h* = −10→10
*T*_min_ = 0.982, *T*_max_ = 0.991	*k* = −10→10
8847 measured reflections	*l* = −16→16

### Refinement

**Table:**

Refinement on *F*^2^	Primary atom site location: structure-invariant direct methods
Least-squares matrix: full	Secondary atom site location: difference Fourier map
*R*[*F*^2^ > 2σ(*F*^2^)] = 0.057	Hydrogen site location: inferred from neighbouring sites
*wR*(*F*^2^) = 0.173	H-atom parameters constrained
*S* = 1.05	*w* = 1/[σ^2^(*F*_o_^2^) + (0.0848*P*)^2^ + 0.2822*P*] where *P* = (*F*_o_^2^ + 2*F*_c_^2^)/3
3391 reflections	(Δ/σ)_max_ < 0.001
209 parameters	Δρ_max_ = 0.36 e Å^−^^3^
0 restraints	Δρ_min_ = −0.25 e Å^−^^3^

### Special details

**Table:**

Geometry. All s.u.'s (except the s.u. in the dihedral angle between two l.s. planes) are estimated using the full covariance matrix. The cell s.u.'s are taken into account individually in the estimation of s.u.'s in distances, angles and torsion angles; correlations between s.u.'s in cell parameters are only used when they are defined by crystal symmetry. An approximate (isotropic) treatment of cell s.u.'s is used for estimating s.u.'s involving l.s. planes.
Refinement. Refinement of *F*^2^ against ALL reflections. The weighted *R*-factor *wR* and goodness of fit *S* are based on *F*^2^, conventional *R*-factors *R* are based on *F*, with *F* set to zero for negative *F*^2^. The threshold expression of *F*^2^ > σ(*F*^2^) is used only for calculating *R*-factors(gt) *etc*. and is not relevant to the choice of reflections for refinement. *R*-factors based on *F*^2^ are statistically about twice as large as those based on *F*, and *R*-factors based on ALL data will be even larger.

### Fractional atomic coordinates and isotropic or equivalent isotropic displacement parameters (Å^2^)

**Table:**

	*x*	*y*	*z*	*U*_iso_*/*U*_eq_	
O1	0.4354 (3)	0.82563 (19)	0.84149 (12)	0.0691 (5)	
O2	0.14909 (19)	1.14174 (17)	0.41431 (11)	0.0546 (4)	
O3	0.1127 (2)	1.37122 (19)	0.24885 (14)	0.0715 (5)	
O4	0.1339 (2)	1.16785 (19)	0.14867 (12)	0.0662 (5)	
C1	0.4303 (3)	0.5733 (2)	0.79154 (15)	0.0462 (5)	
C2	0.5409 (3)	0.4695 (3)	0.86317 (18)	0.0642 (6)	
H2	0.6073	0.5104	0.8967	0.077*	
C3	0.5525 (4)	0.3051 (3)	0.8849 (2)	0.0760 (7)	
H3	0.6276	0.2356	0.9324	0.091*	
C4	0.4536 (4)	0.2448 (3)	0.8364 (2)	0.0731 (7)	
H4	0.4611	0.1347	0.8516	0.088*	
C5	0.3435 (4)	0.3461 (3)	0.7654 (2)	0.0667 (6)	
H5	0.2761	0.3047	0.7331	0.080*	
C6	0.3327 (3)	0.5103 (3)	0.74180 (17)	0.0537 (5)	
H6	0.2600	0.5780	0.6927	0.064*	
C7	0.4101 (3)	0.7530 (2)	0.77353 (15)	0.0476 (5)	
C8	0.3509 (2)	0.8461 (2)	0.67388 (15)	0.0425 (4)	
C9	0.4344 (2)	0.7866 (2)	0.57555 (14)	0.0413 (4)	
C10	0.3660 (2)	0.8849 (2)	0.48841 (14)	0.0432 (4)	
H10	0.4193	0.8466	0.4225	0.052*	
C11	0.2199 (2)	1.0389 (2)	0.49763 (15)	0.0437 (4)	
C12	0.1369 (2)	1.1014 (2)	0.59520 (16)	0.0449 (4)	
C13	0.2055 (3)	1.0032 (2)	0.68133 (15)	0.0457 (4)	
H13	0.1531	1.0430	0.7470	0.055*	
C14	0.6014 (3)	0.6268 (2)	0.56025 (16)	0.0490 (5)	
H14A	0.6369	0.6093	0.4872	0.074*	
H14B	0.5765	0.5326	0.5935	0.074*	
H14C	0.6970	0.6368	0.5902	0.074*	
C15	−0.0212 (3)	1.2694 (2)	0.60437 (19)	0.0596 (6)	
H15A	−0.0484	1.3174	0.5362	0.089*	
H15B	0.0086	1.3446	0.6403	0.089*	
H15C	−0.1241	1.2532	0.6425	0.089*	
C16	0.2201 (3)	1.0816 (3)	0.31397 (16)	0.0523 (5)	
H16A	0.1847	0.9904	0.3029	0.063*	
H16B	0.3507	1.0388	0.3062	0.063*	
C17	0.1474 (3)	1.2266 (3)	0.23580 (17)	0.0519 (5)	
C18	0.0828 (4)	1.2910 (3)	0.06180 (19)	0.0752 (7)	
H18A	0.1834	1.3212	0.0322	0.090*	
H18B	−0.0173	1.3927	0.0856	0.090*	
C19	0.0308 (5)	1.2153 (4)	−0.0163 (2)	0.0967 (10)	
H19A	−0.0037	1.2947	−0.0742	0.145*	
H19B	−0.0689	1.1860	0.0136	0.145*	
H19C	0.1310	1.1153	−0.0398	0.145*	

### Atomic displacement parameters (Å^2^)

**Table:**

	*U*^11^	*U*^22^	*U*^33^	*U*^12^	*U*^13^	*U*^23^
O1	0.1070 (13)	0.0530 (9)	0.0549 (9)	−0.0327 (9)	−0.0290 (9)	−0.0015 (7)
O2	0.0530 (8)	0.0449 (8)	0.0511 (8)	−0.0028 (6)	−0.0123 (6)	0.0043 (6)
O3	0.0911 (12)	0.0458 (9)	0.0748 (11)	−0.0189 (8)	−0.0288 (9)	0.0057 (8)
O4	0.0907 (12)	0.0566 (9)	0.0522 (9)	−0.0281 (8)	−0.0194 (8)	0.0076 (7)
C1	0.0514 (11)	0.0412 (10)	0.0429 (10)	−0.0149 (8)	−0.0053 (8)	−0.0019 (8)
C2	0.0795 (16)	0.0530 (12)	0.0612 (13)	−0.0237 (11)	−0.0233 (12)	0.0062 (10)
C3	0.0950 (19)	0.0504 (13)	0.0734 (16)	−0.0189 (13)	−0.0212 (14)	0.0142 (11)
C4	0.0944 (19)	0.0442 (12)	0.0723 (16)	−0.0254 (12)	0.0105 (14)	−0.0030 (11)
C5	0.0800 (16)	0.0592 (13)	0.0688 (15)	−0.0373 (12)	0.0026 (12)	−0.0127 (11)
C6	0.0568 (12)	0.0522 (11)	0.0534 (12)	−0.0226 (10)	−0.0058 (9)	−0.0036 (9)
C7	0.0524 (11)	0.0426 (10)	0.0468 (11)	−0.0158 (9)	−0.0092 (9)	−0.0043 (8)
C8	0.0451 (10)	0.0354 (9)	0.0472 (10)	−0.0142 (8)	−0.0103 (8)	−0.0026 (7)
C9	0.0379 (9)	0.0346 (9)	0.0494 (10)	−0.0105 (7)	−0.0088 (8)	−0.0029 (7)
C10	0.0412 (10)	0.0391 (9)	0.0437 (10)	−0.0088 (8)	−0.0064 (8)	−0.0038 (8)
C11	0.0412 (9)	0.0384 (9)	0.0489 (11)	−0.0118 (8)	−0.0113 (8)	0.0027 (8)
C12	0.0402 (9)	0.0350 (9)	0.0552 (11)	−0.0092 (8)	−0.0066 (8)	−0.0044 (8)
C13	0.0491 (10)	0.0374 (9)	0.0473 (10)	−0.0132 (8)	−0.0018 (8)	−0.0084 (8)
C14	0.0439 (10)	0.0415 (10)	0.0532 (11)	−0.0060 (8)	−0.0095 (8)	−0.0038 (8)
C15	0.0524 (12)	0.0397 (10)	0.0711 (14)	−0.0017 (9)	−0.0052 (10)	−0.0056 (10)
C16	0.0562 (12)	0.0454 (10)	0.0509 (12)	−0.0142 (9)	−0.0131 (9)	0.0035 (9)
C17	0.0492 (11)	0.0502 (11)	0.0550 (12)	−0.0166 (9)	−0.0143 (9)	0.0045 (9)
C18	0.103 (2)	0.0683 (15)	0.0531 (13)	−0.0326 (15)	−0.0191 (13)	0.0145 (11)
C19	0.130 (3)	0.108 (2)	0.0701 (18)	−0.061 (2)	−0.0400 (18)	0.0194 (16)

### Geometric parameters (Å, °)

**Table:**

O1—C7	1.222 (2)	C9—C14	1.508 (2)
O2—C11	1.373 (2)	C10—C11	1.389 (3)
O2—C16	1.408 (3)	C10—H10	0.9300
O3—C17	1.190 (3)	C11—C12	1.397 (3)
O4—C17	1.327 (3)	C12—C13	1.382 (3)
O4—C18	1.459 (3)	C12—C15	1.510 (3)
C1—C6	1.387 (3)	C13—H13	0.9300
C1—C2	1.389 (3)	C14—H14A	0.9600
C1—C7	1.491 (3)	C14—H14B	0.9600
C2—C3	1.387 (3)	C14—H14C	0.9600
C2—H2	0.9300	C15—H15A	0.9600
C3—C4	1.370 (4)	C15—H15B	0.9600
C3—H3	0.9300	C15—H15C	0.9600
C4—C5	1.375 (4)	C16—C17	1.512 (3)
C4—H4	0.9300	C16—H16A	0.9700
C5—C6	1.390 (3)	C16—H16B	0.9700
C5—H5	0.9300	C18—C19	1.461 (4)
C6—H6	0.9300	C18—H18A	0.9700
C7—C8	1.494 (3)	C18—H18B	0.9700
C8—C9	1.400 (3)	C19—H19A	0.9600
C8—C13	1.402 (3)	C19—H19B	0.9600
C9—C10	1.392 (3)	C19—H19C	0.9600
			
C11—O2—C16	117.92 (15)	C11—C12—C15	120.57 (18)
C17—O4—C18	116.19 (18)	C12—C13—C8	122.77 (18)
C6—C1—C2	119.25 (19)	C12—C13—H13	118.6
C6—C1—C7	120.90 (18)	C8—C13—H13	118.6
C2—C1—C7	119.76 (18)	C9—C14—H14A	109.5
C3—C2—C1	120.2 (2)	C9—C14—H14B	109.5
C3—C2—H2	119.9	H14A—C14—H14B	109.5
C1—C2—H2	119.9	C9—C14—H14C	109.5
C4—C3—C2	120.1 (2)	H14A—C14—H14C	109.5
C4—C3—H3	119.9	H14B—C14—H14C	109.5
C2—C3—H3	119.9	C12—C15—H15A	109.5
C3—C4—C5	120.3 (2)	C12—C15—H15B	109.5
C3—C4—H4	119.8	H15A—C15—H15B	109.5
C5—C4—H4	119.8	C12—C15—H15C	109.5
C4—C5—C6	120.1 (2)	H15A—C15—H15C	109.5
C4—C5—H5	119.9	H15B—C15—H15C	109.5
C6—C5—H5	119.9	O2—C16—C17	108.12 (16)
C1—C6—C5	120.0 (2)	O2—C16—H16A	110.1
C1—C6—H6	120.0	C17—C16—H16A	110.1
C5—C6—H6	120.0	O2—C16—H16B	110.1
O1—C7—C1	120.15 (18)	C17—C16—H16B	110.1
O1—C7—C8	120.08 (17)	H16A—C16—H16B	108.4
C1—C7—C8	119.71 (16)	O3—C17—O4	124.87 (19)
C9—C8—C13	119.31 (17)	O3—C17—C16	125.4 (2)
C9—C8—C7	123.67 (16)	O4—C17—C16	109.72 (18)
C13—C8—C7	117.01 (17)	O4—C18—C19	108.2 (2)
C10—C9—C8	118.21 (16)	O4—C18—H18A	110.1
C10—C9—C14	118.84 (17)	C19—C18—H18A	110.1
C8—C9—C14	122.83 (16)	O4—C18—H18B	110.1
C11—C10—C9	121.49 (17)	C19—C18—H18B	110.1
C11—C10—H10	119.3	H18A—C18—H18B	108.4
C9—C10—H10	119.3	C18—C19—H19A	109.5
O2—C11—C10	123.79 (17)	C18—C19—H19B	109.5
O2—C11—C12	115.21 (16)	H19A—C19—H19B	109.5
C10—C11—C12	120.99 (17)	C18—C19—H19C	109.5
C13—C12—C11	117.21 (16)	H19A—C19—H19C	109.5
C13—C12—C15	122.22 (18)	H19B—C19—H19C	109.5
			
C6—C1—C2—C3	−0.3 (4)	C8—C9—C10—C11	0.6 (3)
C7—C1—C2—C3	176.3 (2)	C14—C9—C10—C11	−175.62 (17)
C1—C2—C3—C4	−0.6 (4)	C16—O2—C11—C10	4.9 (3)
C2—C3—C4—C5	0.5 (4)	C16—O2—C11—C12	−176.40 (17)
C3—C4—C5—C6	0.4 (4)	C9—C10—C11—O2	179.02 (17)
C2—C1—C6—C5	1.3 (3)	C9—C10—C11—C12	0.4 (3)
C7—C1—C6—C5	−175.3 (2)	O2—C11—C12—C13	−179.07 (16)
C4—C5—C6—C1	−1.3 (3)	C10—C11—C12—C13	−0.4 (3)
C6—C1—C7—O1	151.5 (2)	O2—C11—C12—C15	1.0 (3)
C2—C1—C7—O1	−25.1 (3)	C10—C11—C12—C15	179.66 (18)
C6—C1—C7—C8	−25.8 (3)	C11—C12—C13—C8	−0.7 (3)
C2—C1—C7—C8	157.6 (2)	C15—C12—C13—C8	179.29 (18)
O1—C7—C8—C9	130.0 (2)	C9—C8—C13—C12	1.7 (3)
C1—C7—C8—C9	−52.7 (3)	C7—C8—C13—C12	−179.52 (17)
O1—C7—C8—C13	−48.7 (3)	C11—O2—C16—C17	−169.54 (16)
C1—C7—C8—C13	128.6 (2)	C18—O4—C17—O3	3.8 (3)
C13—C8—C9—C10	−1.6 (3)	C18—O4—C17—C16	−174.0 (2)
C7—C8—C9—C10	179.72 (16)	O2—C16—C17—O3	32.4 (3)
C13—C8—C9—C14	174.45 (17)	O2—C16—C17—O4	−149.82 (18)
C7—C8—C9—C14	−4.3 (3)	C17—O4—C18—C19	−165.9 (2)

### Hydrogen-bond geometry (Å, °)

**Table:**

*D*—H···*A*	*D*—H	H···*A*	*D*···*A*	*D*—H···*A*
C15—H15A···O2	0.96	2.28	2.751 (3)	110
